# A rotavirus VP4 or VP7 monoreassortant panel identifies genotypes that are less susceptible to neutralization by systemic antibodies induced by vaccination or natural infection

**DOI:** 10.1128/mbio.00897-25

**Published:** 2025-05-30

**Authors:** Tomohiro Kotaki, Yuta Kanai, Kristen M. Ogden, Megumi Onishi, Kattareeya Kumthip, Pattara Khamrin, Patcharaporn Boonyos, Pornkamol Phoosangwalthong, Phakapun Singchai, Tipsuda Luechakham, Shohei Minami, Zelin Chen, Katsuhisa Hirai, Ratana Tacharoenmuang, Hiroto Mizushima, Hiroshi Ushijima, Niwat Maneekarn, Takeshi Kobayashi

**Affiliations:** 1 Department of Virology, Research Institute for Microbial Diseases, The University of Osaka 34822 , Osaka, Japan; 2 Department of Pediatrics, Vanderbilt University Medical Center 12328, Nashville, Tennessee, USA; 3 Department of Microbiology, Faculty of Medicine, Chiang Mai University 627138, Chiang Mai, Thailand; 4 Center of Excellence in Emerging and Re-emerging Diarrheal Viruses, Chiang Mai University 26682, Chiang Mai, Thailand; 5 Thailand-Japan Research Collaboration Center on Emerging and Re-emerging Infections 13013, Nonthaburi, Thailand; 6 Department of Medical Sciences, Ministry of Public Health, Nonthaburi, Thailand; 7 Department of Pathology and Microbiology, Nihon University School of Medicine 625041 , Tokyo, Japan; 8 Center for Infectious Disease Education and Research, The University of Osaka 13013, Osaka, Japan; 9 Center for Advanced Modalities and DDS, The University of Osaka 13013, Osaka, Japan; St. Jude Children's Research Hospital, Memphis, Tennessee, USA; Instituto de Biotecnología, UNAM, Cuernavaca, Morelos, Mexico

**Keywords:** rotavirus, VP4, VP7, genotype, reverse genetics, neutralization susceptibility, vaccine

## Abstract

Rotavirus infections remain a significant cause of morbidity and mortality in infants. The viral surface proteins VP4 and VP7 are each classified into multiple genotypes (P[1]–P[58] for VP4 and G1–G42 for VP7), which differ in their susceptibility to neutralizing antibodies; however, detailed analyses of these differences remain limited. This study investigates the susceptibility of diverse VP4 and VP7 genotypes to neutralizing antibodies induced by vaccination or natural infection. A reverse genetics system based on the human rotavirus Odelia strain (G4P[8]) was used to create monoreassortant viruses by replacing the VP4 or VP7 genes with those from clinical isolates (obtained in Thailand) or prototype strains. We generated 11 VP4 and 19 VP7 reassortants, covering 7 and 17 genotypes, respectively. Neutralization tests using the monoreassortant viruses, immune sera, and MA104 cells indicate that the VP7 genotype, rather than the VP4 genotype, primarily influences susceptibility to neutralization. Furthermore, genotypes G2, bat-like G3, G11, G12, G25, and G33 were significantly less susceptible to sera from naturally infected Thai individuals, highlighting the importance of monitoring these genotypes. To identify the protein region responsible for susceptibility to neutralization, we generated chimeric viruses by exchanging the domains of high- and low-susceptibility VP7 genotypes and found that Domain I appears to play a substantial role in determining susceptibility. Thus, we identified multiple rotavirus genotypes with reduced susceptibility to neutralization, as well as the region primarily responsible for this reduction. These findings may help to predict possible future rotavirus outbreaks in humans and facilitate the development of effective rotavirus vaccines.

**IMPORTANCE:**

Rotavirus, the leading cause of severe acute gastroenteritis in infants, is responsible for approximately 128,500 infant deaths globally each year. The virus’s surface proteins are highly diverse, comprising approximately 100 genotypes. This diversity affects susceptibility to neutralizing antibodies and the efficacy of vaccines. Here, we found that certain genotypes are highly resistant to serum neutralizing antibodies induced by vaccination and natural infections. Furthermore, we identified a specific region in the viral outer capsid protein that plays a significant role in determining susceptibility to neutralization. These findings may be important for predicting outbreak-causing strains and for developing more effective vaccines, ultimately contributing to the prevention of future outbreaks and improving global infant health.

## INTRODUCTION

Rotavirus is a leading cause of severe gastroenteritis, particularly in infants and young children (1). Rotavirus vaccines are widely available and have reduced the burden of rotavirus infections substantially; however, rotavirus remains a major public health concern, causing approximately 128,500 deaths annually worldwide (2). Thus, there is a pressing need to improve prevention strategies.

Rotavirus is a non-enveloped, double-stranded RNA (dsRNA) virus that belongs to the family *Sedoreoviridae* (3). Its genome comprises 11 segments encoding six structural proteins (VP1–VP4, VP6, and VP7) and six non-structural proteins (NSP1–NSP6) (4). Among the structural proteins, VP4 and VP7 are located on the virion surface (5–7). VP4 is a spike protein that mediates attachment of the virus to susceptible cells (8), while VP7, a glycoprotein that forms the outer capsid, mediates cell entry by interacting with coreceptors (9). These proteins are the primary determinants of viral infectivity and are major targets of neutralizing antibodies (10).

VP4 and VP7 are highly diverse and comprise multiple genotypes: VP4 defines P genotypes, ranging from P[1] to P[58], while VP7 defines G genotypes, ranging from G1 to G42 (as of March 2025) (11, 12). In general, the genotype of a rotavirus is expressed as a combination of its VP7 and VP4 genotypes (e.g., the genotype of human rotavirus Odelia strain is G4P[8]). According to the Rotavirus Classification Working Group (RCWG), the nucleotide identity cut-off value defining genotypes for VP4 and VP7 is 80% (13). These genetic differences can affect viral replication and susceptibility to neutralizing antibodies, both of which play important roles in determining the viral strains that emerge and spread within populations (14–16). Although several neutralizing epitopes have been identified (5, 17), the impact of genotype on susceptibility to neutralization, and the regions of VP4 and VP7 responsible for determining susceptibility to neutralization, remain unclear.

Rotavirus infects a wide range of hosts, including pigs, horses, birds, and bats (18–21). The host range is influenced primarily by the G and P genotypes. For example, human rotaviruses commonly have genotypes G1P[8], G2P[4], G3P[8], G4P[8], G9P[8], or G12P[8] (22); however, cross-species transmission has been reported (22–24). Importantly, zoonotic transmission can facilitate the generation of novel reassortant rotaviruses (22–24); indeed, the equine-like G3 genotype, likely originating from horses, was identified during a human outbreak and has become prevalent in many countries (25, 26). These events highlight the importance of monitoring non-human rotavirus genotypes and their potential to reassort with human rotaviruses, as well as the need to understand the mechanisms used by these viruses to evade human immune responses.

Currently, two live-attenuated rotavirus vaccines, Rotarix (monovalent G1P[8]) and RotaTeq (a pentavalent vaccine comprising G1P[5], G2P[5], G3P[5], G4P[5], and G6P[8]), are used widely and have reduced the burden of rotavirus infections substantially (27–29); however, their efficacy varies by region, possibly due in part to differences in circulating genotypes (30–34). Several studies show that mass vaccination programs may change the genotype distribution of circulating rotavirus, although this is still controversial (35–41). For example, the G1P[8] genotype, which is homologous to the Rotarix vaccine, has become less prevalent following the introduction of vaccination programs (37–39). By contrast, the G2P[4] and G12P[8] genotypes, which were less common before the introduction of vaccination programs, have become more prevalent in many countries, indicating that certain genotypes may escape immunity induced by current vaccines (36, 38–41). Considering the above situations, understanding how different genotypes affect viral replication and susceptibility to neutralizing antibodies is critical for improving vaccine efficacy and predicting potential outbreaks.

Reverse genetics systems are useful for studying the effects of different genotypes on viral replication and susceptibility to neutralizing antibodies (42). These systems enable us to generate monoreassortant viruses harboring the VP4 or VP7 segment of various rotavirus strains. Previously, we reported that altering VP4 or VP7 genotypes affects induction of/susceptibility to neutralizing antibodies (14). Additionally, we reported the susceptibility of multiple VP7 genotypes to antibodies induced by rotavirus vaccines (15); however, those studies focused on common human rotavirus genotypes rather than non-human rotavirus genotypes. Understanding the effects of genotype differences in both human and non-human rotaviruses is important to predict possible future outbreaks in humans and for more effective vaccine design.

Here, we examined the impact of genotype differences on the susceptibility of rotavirus to neutralization. Using a reverse genetics system, we generated VP4 or VP7 monoreassortant viruses based on sequence data from human rotavirus clinical isolates, as well as various prototype strains from both human and non-human rotavirus genotypes. We then investigated the susceptibility of these recombinant viruses to systemic neutralizing antibodies induced by vaccination or natural infection. We identified several genotypes that are less susceptible to neutralizing antibodies, as well as the region primarily responsible for this reduction in susceptibility. These findings may enable us to better predict possible future outbreaks in humans, as well as develop more effective rotavirus vaccines.

## RESULTS

### Generation of VP4 or VP7 monoreassortant viruses harboring genes derived from clinical isolates

**Fig 1:**
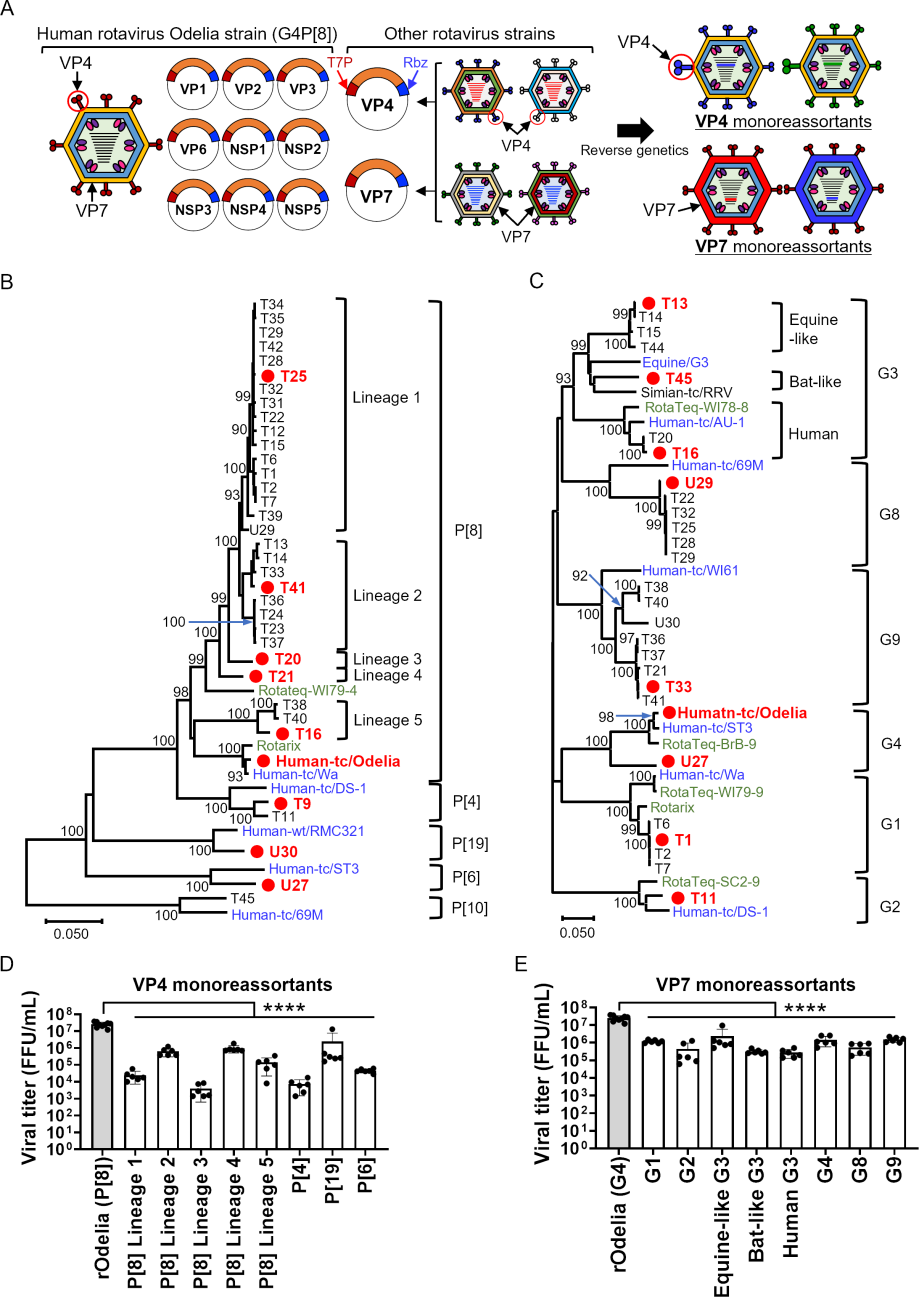
Generation of monoreassortant viruses harboring VP4 or VP7 derived from clinical isolates. (**A**) Schematic diagram showing generation of monoreassortant viruses. Each viral genome was cloned downstream of the T7 promoter (T7P) and linked to the hepatitis D virus ribozyme (Rbz). (**B, C**) Phylogenetic analyses of the VP4 (**B**) and VP7 (**C**) genes derived from clinical samples. The Thai clinical isolates sequenced in this study are represented by their ID numbers, starting with T or U followed by one or two digits. The isolates highlighted in red within a red circle were used for monoreassortant virus generation. Prototype and vaccine strains are highlighted in blue and green, respectively. Prototype strains are shown in the format: origin species/strain name. (**D, E**) Growth of monoreassortant viruses containing VP4 (**D**) or VP7 (**E**) with the indicated genotype from a Thai clinical isolate. MA104 cells were infected with viruses at an MOI of 0.001 and harvested at 72 h post-infection. Statistical significance was analyzed using one-way ANOVA with Dunnett’s multiple comparison test. *P* values < 0.05 were considered statistically significant. ****P value < 0.0001.

To analyze the effect of rotavirus genotypes on viral replication and susceptibility to neutralizing antibodies, we first generated VP4 or VP7 monoreassortant viruses harboring genes from currently circulating human rotaviruses (Fig. 1A). RNA was extracted from human fecal samples collected in Chiang Mai, Thailand, from 2010 to 2020 (18, 43–45). The viral VP4 or VP7 segments were amplified by PCR, cloned into plasmids, and sequenced. At least one of the genes from each of 36 samples was sequenced (Table 1). Representative strains are shown in the phylogenetic trees (Fig. 1B and C). The G9P[8] (27.7%; 10/36) genotype was the most dominant, followed by G8P[8] (25.0%; 9/36), G3P[8] (13.9%; 5/36), G1P[8] (11.1%; 4/36), and G2P[4] (2.8%; 1/36). These genotypes are common in humans in Thailand. Over the past two decades, the G1P[8], G2P[4], G3P[8], G8P[8], and G9P[8] genotypes have been prevalent in Thailand (43, 46). In addition, the G3P[10], G4P[6], and G9P[19] genotypes, which are not usually prevalent in humans, were detected. The G3P[10] (virus ID: T45) may be derived from bats, while G4P[6] (U27) and G9P[19] (U30) are derived from pigs (18, 44, 45).

**TABLE 1:** Epidemiological information for the clinical isolates

Genotype	ID	Strain name	Year of isolation
G1P[8]	T1	CMH-S119-19	2019
	T2	CMH-S008-18	2018
	T6	CMH-N084-18	2018
	T7	CMH-N085-18	2018
G2P[4]	T11	CMH-S118-18	2018
Equine-like G3P[8]	T13	CMH-ST019-20	2020
	T14	CMH-ST030-20	2020
	T15	CMH-S049-19	2019
Human G3P[8]	T16	CMH-S152-19	2019
	T20	CMH-ST230-19	2018
G8P[8]	T22	CMH-R057-19	2019
	T25	CMH-S022-18	2018
	T28	CMH-N030-18	2018
	T29	CMH-N055-18	2018
	T32	CMH-ST137-18	2018
	T34	CMH-ST022-20	2020
	T35	CMH-ST025-20	2020
	T42	CMH-S081-18	2018
	U29	CMH-N165-13	2013
G9P[8]	T21	CMH-R035-19	2019
	T23	CMH-R146-19	2019
	T24	CMH-R180-19	2019
	T31	CMH-ST077-18	2018
	T33	CMH-S011-20	2020
	T36	CMH-R137-19	2019
	T37	CMH-R154-19	2019
	T38	CMH-S002-18	2018
	T40	CMH-S042-18	2018
	T41	CMH-S069-18	2018
Bat-like G3P[10]	T45	CMH-S015-19	2019
G4P[6]	U27	CMH-N014-11	2011
G9P[19]	U30	CMH-S070-13	2013
Equine-like G3P[x]	T44	CMH-ST208-18	2018
GxP[4]	T9	CMH-S006-20	2020
GxP[8]	T12	CMH-ST067-18	2018
	T39	CMH-S007-18	2018

Next, constructed plasmids were used to generate VP4 or VP7 monoreassortant viruses. In this study, we chose the human rotavirus Odelia strain as a backbone rather than the simian rotavirus SA11 strain that is commonly used in rotavirus reverse genetics studies (47). We expected that monoreassortant viruses generated using the Odelia strain backbone would more closely resemble naturally circulating human rotavirus strains than those generated using the simian rotavirus SA11 strain backbone. In addition, the reverse genetics system based on the Odelia strain enabled us to evaluate the potential for certain genotypes to reassort with human rotavirus.

The VP4 or VP7 genes used to generate recombinant viruses were selected based on phylogenetic distance (Fig. 1B and C). Due to the high diversity of the P[8] and G3 genotypes, we further divided them into five lineages (P[8] Lineages 1–5) and three sub-genotypes (equine-like G3, bat-like G3, and human G3), respectively (Fig. 1B and C). The bat-like G3 genotype was so named because it originates from the bat-like G3P[10] strain (44). A total of eight VP4 reassortants covering the P[8], P[4], P[19], and P[6] genotypes, and eight VP7 reassortants covering the G1, G2, G3, G4, G8, and G9 genotypes were rescued successfully. The monoreassortant virus harboring the P[10] genotype derived from the bat-like G3P[10] strain was not rescued, despite multiple attempts. In general, replication of the reassortant viruses was significantly lower than that of the parental recombinant Odelia (rOdelia) strain (Fig. 1D and E). Replication of some monoreassortant viruses, particularly those harboring the P[8] Lineage 1, P[8] Lineage 3, P[4], and P[6] genotypes, was >100-fold lower than that of the parental rOdelia strain.

### Generation of VP4 or VP7 monoreassortant viruses harboring genes derived from prototype strains

**Fig 2:**
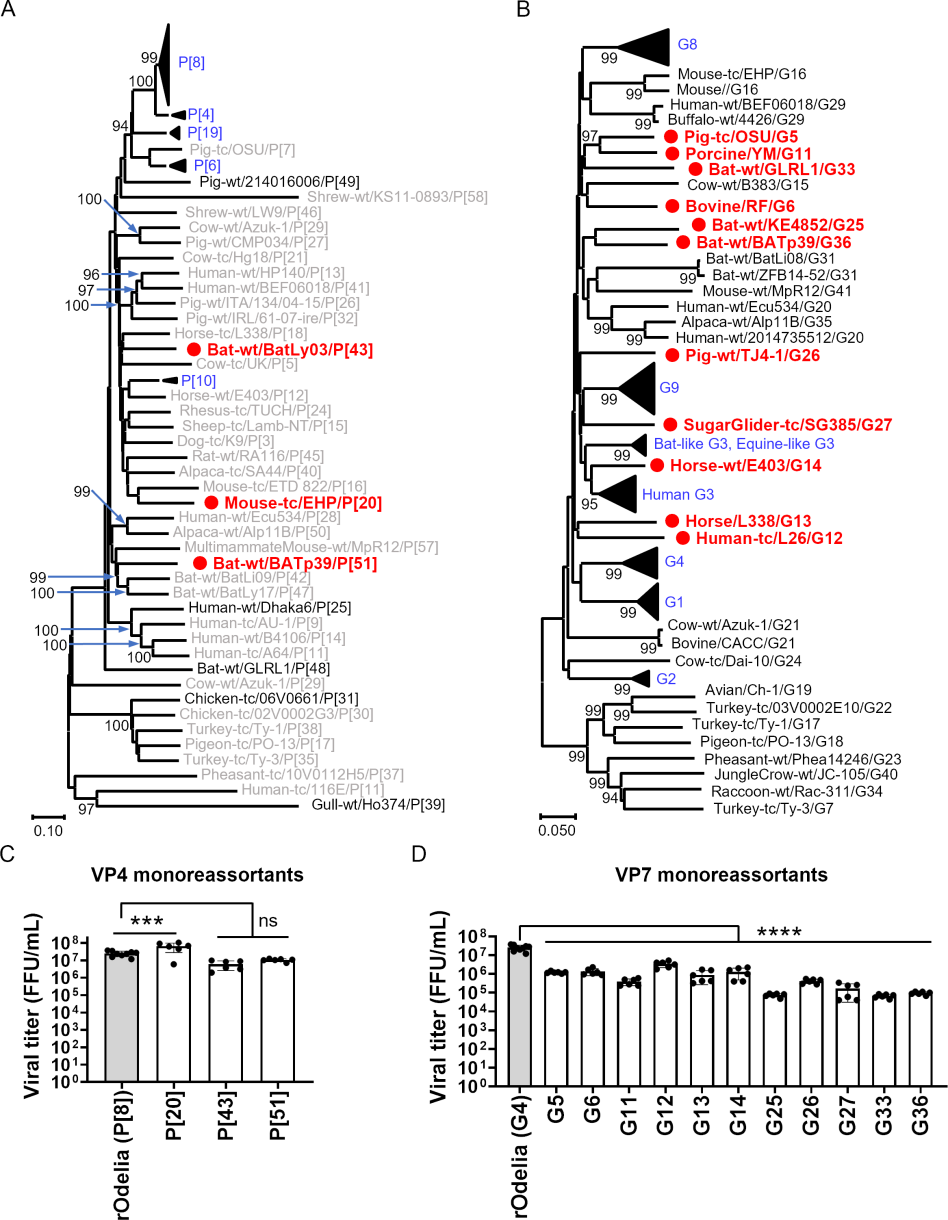
Generation of monoreassortant viruses harboring VP4 or VP7 derived from prototype strains. (**A, B**) Phylogenetic analyses of the VP4 (**A**) and VP7 (**B**) genes. Prototype strains are shown in the format: origin species/strain name/genotype. Prototype strains that were not synthesized are shown in gray. Strains that were synthesized, but for which generation of monoreassortant viruses was unsuccessful, are shown in black. Strains that were synthesized and successfully used to rescue monoreassortant viruses are highlighted in red with a red circle. Genotypes detected in human clinical samples are highlighted in blue. (**C, D**) Growth of monoreassortant viruses containing VP4 (**C**) or VP7 (**D**) of the indicated genotype. MA104 cells were infected with viruses at an MOI of 0.001 and harvested at 72 h post-infection. Statistical significance was analyzed using one-way ANOVA with Dunnett’s multiple comparison test. *P* values < 0.05 were considered statistically significant. ****P value < 0.0001. ns = not significant.

We successfully rescued rotaviruses harboring VP4 or VP7 from commonly prevalent human rotaviruses; however, it is possible that genotypes from non-human rotaviruses, which could potentially escape human immunity, may emerge and cause outbreaks in humans. Therefore, the genes of prototype strains with genotypes not detected in our cohort were synthesized. Selection of the prototype strains used for this was based on a list of newly assigned genotypes provided by the RCWG (11, 12). A total of nine VP4 sequences covering nine genotypes (P[20], P[25], P[31], P[32], P[39], P[43], P[48], P[49], and P[51]) were synthesized. Selection of these genotypes was based primarily on their phylogenetic distance (Fig. 2A). In addition, we synthesized 33 VP7 sequences covering most genotypes available in the database (G5, G6, G7, G11, G12, G13, G14, G15, G16, G17, G18, G19, G20, G21, G22, G23, G24, G25, G26, G27, G29, G31, G33, G34, G35, G36, G40, and G41). The G10, G28, G30, G32, G37, G38, G39, and G42 genotypes were not synthesized due to their incomplete open reading frame sequences, or for practical reasons. The phylogenetic distance of these strains was then examined (Fig. 2B).

Three VP4 monoreassortants (P[20], P[43], and P[51]) and 11 VP7 monoreassortants (G5, G6, G11, G12, G13, G14, G25, G26, G27, G33, and G36) were rescued successfully. Replication of some monoreassortant viruses, particularly those harboring the G25, G33, and G36 genotypes, was >100-fold lower than that of the parental rOdelia strain (Fig. 2C and D). Rescue of these monoreassortant viruses is summarized in Table S1.

### Susceptibility of viruses to antibodies induced by immunization with Rotarix or RotaTeq

In total, we generated 11 VP4 monoreassortants and 19 VP7 monoreassortants, covering 7 and 17 genotypes, respectively. This panel of recombinant viruses was useful for analyzing susceptibility to neutralizing antibodies.

**Fig 3:**
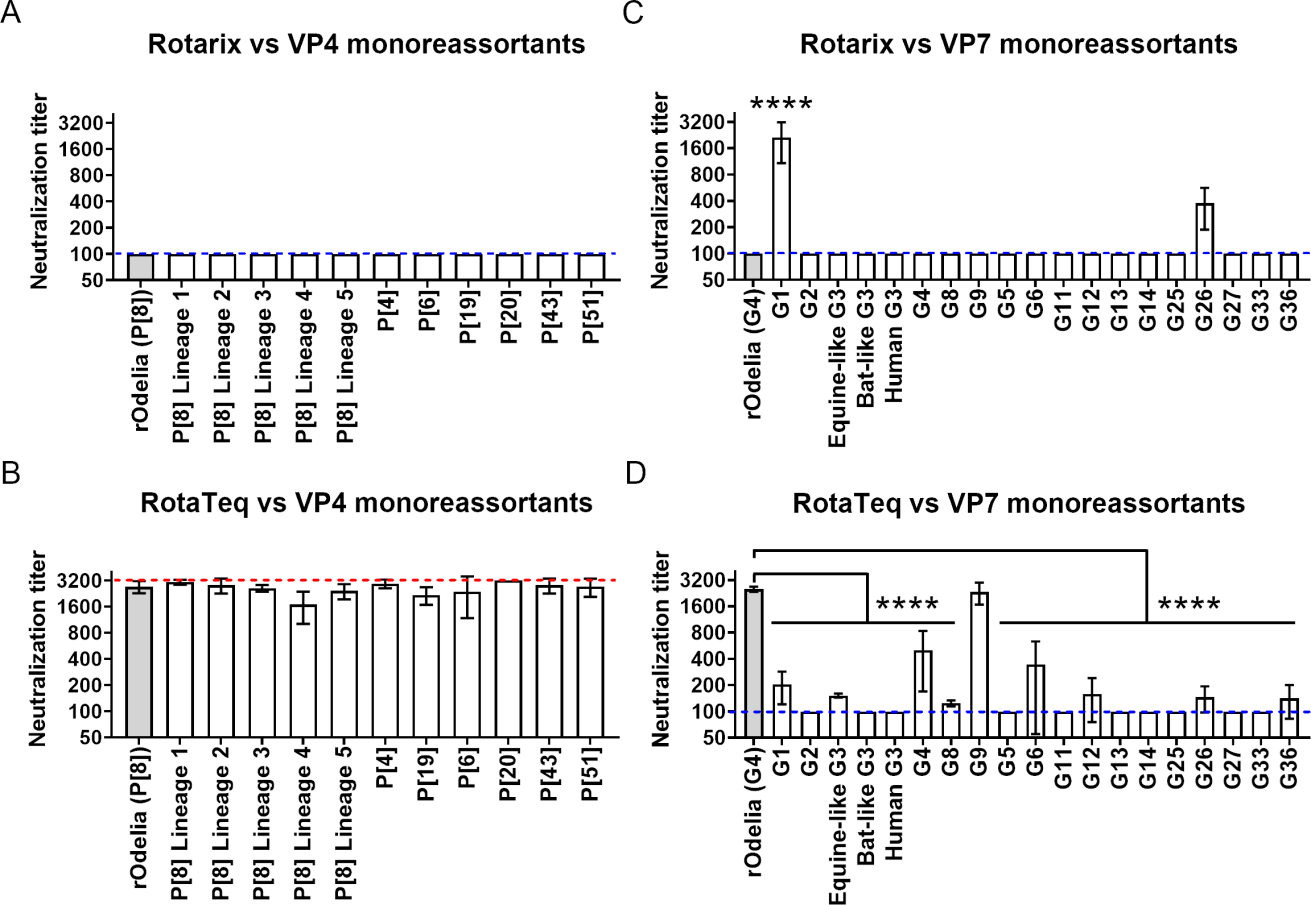
Susceptibility of viruses to Rotarix- or RotaTeq-immunized rabbit serum. (**A, B**) Neutralization test using VP4 monoreassortant viruses and Rotarix- or RotaTeq-immunized rabbit serum. (**C, D**) Neutralization test using VP7 monoreassortant viruses and Rotarix- or RotaTeq-immunized rabbit serum. The neutralization titer is expressed as the serum dilution yielding a 50% reduction in infected cells. The blue dotted line represents the lower limit of detection (1:100), and the red dotted line represents the upper limit of detection (1:3,200). Neutralization titers < 1:100 or >1:3,200 are plotted at the detection limit lines. Statistical significance compared to rOdelia was analyzed using one-way ANOVA with Dunnett’s multiple comparison test. *P* values < 0.05 were considered statistically significant and shown in the figure. ****P value < 0.0001.

First, we analyzed the susceptibility of these viruses to serum antibodies induced by vaccination with Rotarix or RotaTeq, two commonly used rotavirus vaccines (27, 28). Antisera were prepared by immunizing rabbits (two times via the intramuscular route) with these vaccines (15). MA104 cells, the standard used in the field, were used for the neutralization tests. rOdelia strain (G4P[8]) was neutralized by antibodies induced by RotaTeq, but not by those induced by Rotarix. Susceptibility of the VP4 reassortants to neutralization was similar to that of the parental rOdelia strain (Fig. 3A and B). Neutralization of all VP4 monoreassortants by Rotarix-immunized serum was inefficient (NT_50_ < 1:100); however, they were highly susceptible to RotaTeq-immunized serum. The differences between rOdelia and the VP4 monoreassortants were within twofold. This suggests that VP4 does not have a marked effect on susceptibility to neutralization.

By contrast, VP7 reassortants exhibited highly variable susceptibility to neutralization depending on their genotype (Fig. 3C and D). Most VP7 monoreassortants were not susceptible to antibodies induced by Rotarix. By contrast, the G1 genotype was highly susceptible to Rotarix-immunized rabbit serum, which is expected because Rotarix is a G1P[8] genotype vaccine. VP7 monoreassortants harboring the G1, Equine-like G3, G4, G8, G9, G6, G12, G26, or G36 genotypes were neutralized (NT_50_ > 1:100) by RotaTeq-immunized rabbit serum (Fig. 3D). It is noteworthy that non-vaccine genotypes (G8, G9, G12, G26, and G36) were cross-neutralized. To summarize, VP7 genotypes rather than VP4 genotypes largely influence rotavirus susceptibility to neutralizing antibodies induced by immunization.

### Susceptibility to antibodies induced by natural rotavirus infection

**Fig 4:**
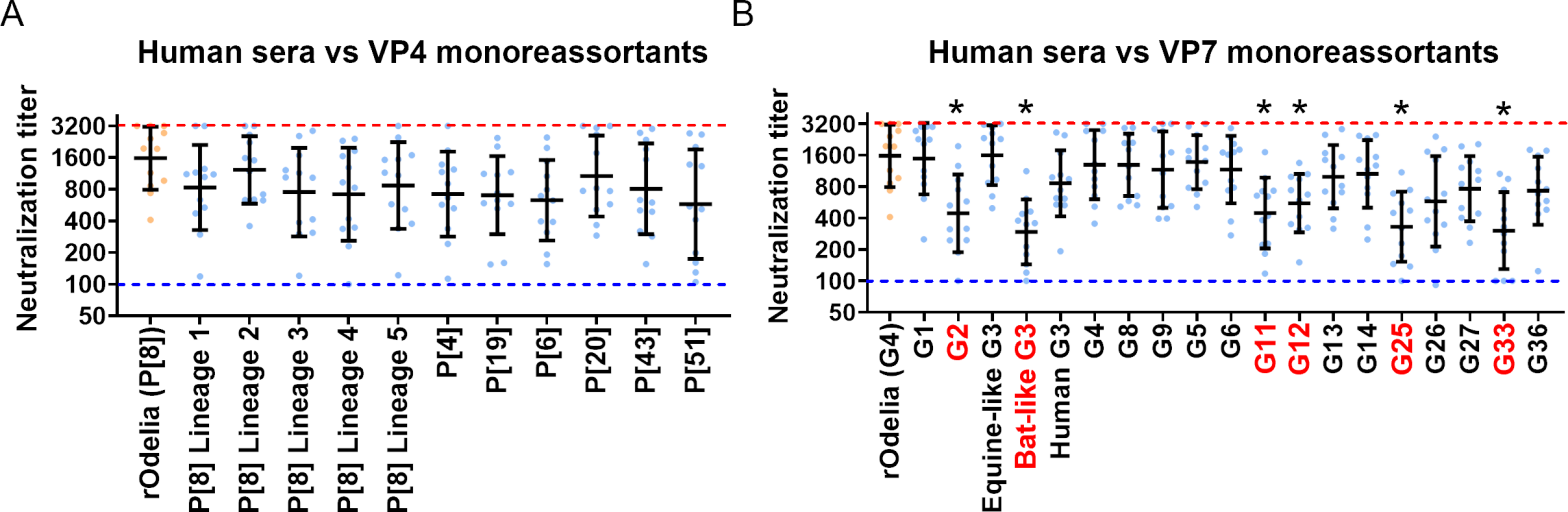
Susceptibility of the viruses to neutralization by serum from rotavirus-infected humans. (**A, B**) Neutralization test using VP4 (**A**) or VP7 (**B**) monoreassortant viruses and human sera. The neutralization titer is expressed as the serum dilution yielding a 50% reduction in infected cells. The blue dotted line indicates the lower limit of detection (1:100), and the red dotted line indicates the upper limit of detection (1:3,200). Neutralization titers <1:100 or >1:3,200 are plotted at the detection limit lines. Each circle represents serum from an individual (*n* = 12). A substantial reduction in neutralization, denoted by *, was defined by the following criteria: (i) *P* < 0.0001 by Friedman test (compared with rOdelia), and (ii) a >1.5 log2-fold change in the geometric mean titer (compared with rOdelia).

Next, we analyzed susceptibility to neutralization by antibodies induced by natural rotavirus infection. Human sera were collected from healthy Thai adults (31–58 years old) who had not received a rotavirus vaccination. A total of 12 serum samples from 12 individuals were used for the neutralization test. All sera showed neutralizing titers >1:100 against the parental rOdelia strain, indicating that all individuals had been exposed to a previous rotavirus infection (Fig. 4). Due to the high variation in neutralizing titers among individuals, we defined a marked reduction in neutralization using the following criteria: (i) *P* < 0.0001 by Friedman test (compared with rOdelia), and (ii) a >1.5 log_2_-fold change in the geometric mean titer (compared with rOdelia).

None of the VP4 monoreassortants exhibited a marked reduction in susceptibility to neutralization (Fig. 4A), a finding consistent with the results obtained using vaccinated rabbit sera (Fig. 3A and B). By contrast, VP7 monoreassortants harboring the G2, bat-like G3, G11, G12, G25, and G33 genotypes exhibited reduced susceptibility to neutralization (Fig. 4B), suggesting that these genotypes may have the potential to escape pre-existing antibodies induced by naturally circulating rotavirus in Thailand, potentially allowing them to become dominant circulating strains.

### Domain I of VP7 is a determinant of susceptibility to neutralizing antibodies

**Fig 5:**
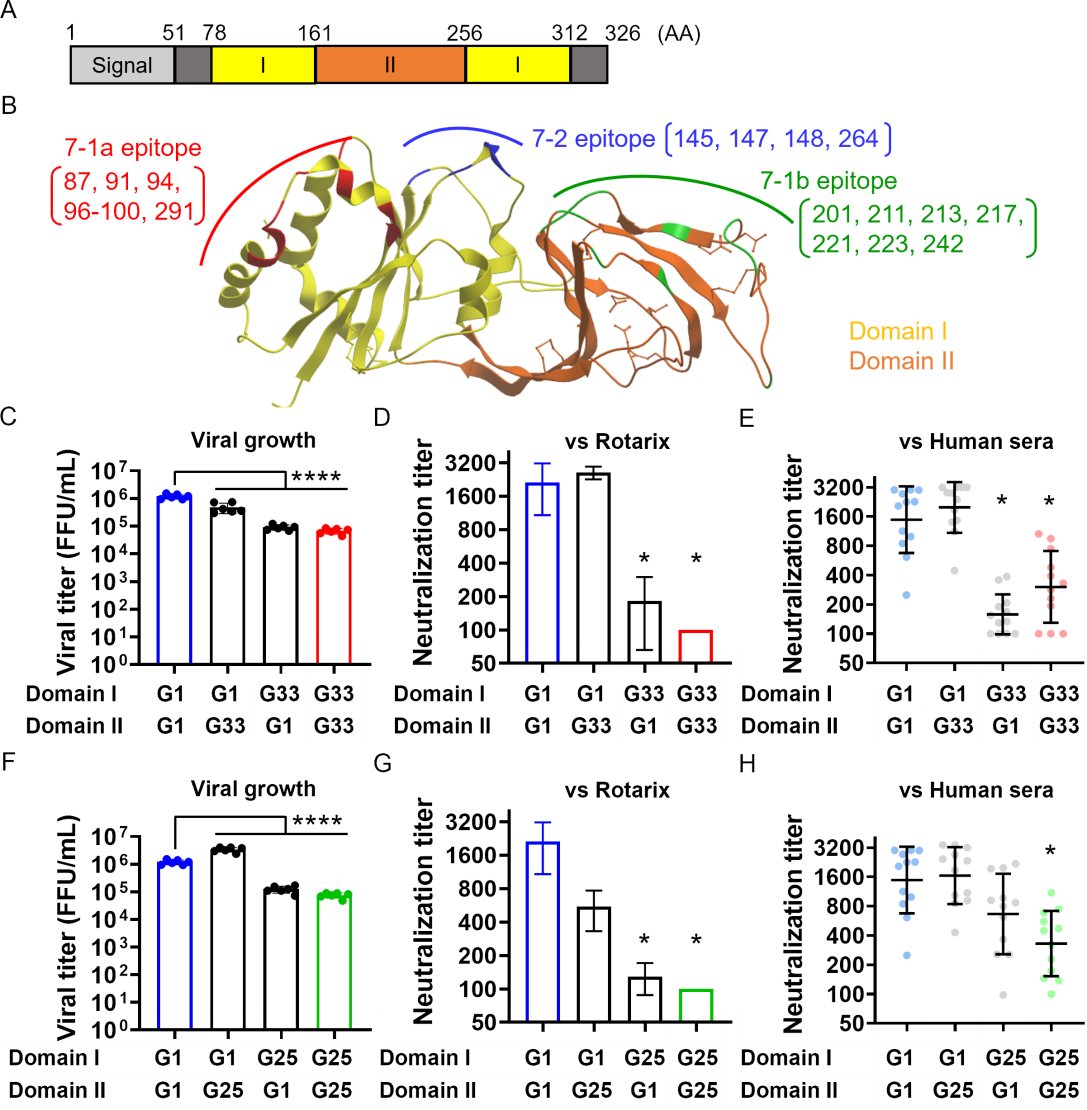
Domain I of VP7 is a determinant of susceptibility to neutralization. (**A**) Linear schematic showing the domain organization of the VP7 protein. The number of amino acid residues is shown above. (**B**) Structure of the VP7 protein. The ribbon diagram is based on Protein Data Bank (PDB) accession number 3FMG. Domain I and Domain II are colored yellow and orange, respectively. Critical amino acid residues within the 7-1a, 7-1b, and 7-2 epitopes are highlighted in red, green, and blue, respectively. (**C**) Growth of the G1-G33 chimeric viruses. MA104 cells were infected with the viruses at an MOI of 0.001 and harvested at 72 h post-infection. Statistical significance was analyzed using one-way ANOVA with Dunnett’s multiple comparison test. *P* values < 0.05 were considered statistically significant. ****P value < 0.0001. (**D**) Susceptibility of the G1–G33 chimeric viruses to neutralization by Rotarix-immunized serum. Statistical significance was analyzed using one-way ANOVA with Dunnett’s multiple comparison test. *P* values < 0.05 were considered statistically significant and shown in the figure. *P value < 0.05. (**E**) Susceptibility of the G1–G33 chimeric viruses to neutralization by human sera. Each circle represents serum from an individual (*n* = 12). A substantial reduction in neutralization, denoted by *, was defined by the following criteria: (i) *P* < 0.0001 by Friedman test (compared with the G1 monoreassortant), and (ii) a >1.5 log2-fold change in the geometric mean titer (compared with the G1 monoreassortant). (**F**) Growth of the G1–G25 chimeric viruses. (**G**) Susceptibility of the G1–G25 chimeric viruses to neutralization by Rotarix-immunized serum. (**H**) Susceptibility of the G1–G25 chimeric viruses to neutralization by human sera.

We identified several genotypes that affect susceptibility to neutralizing antibodies (Table 2). There was a clear difference in NT_50_ values between the high-susceptibility and low-susceptibility genotypes; however, neither amino acid comparisons nor phylogenetic analysis revealed distinct differences or conserved epitope regions between the two groups (Fig. S1 and S2). Therefore, we dissected the determinants of VP7 that affect susceptibility to neutralization through structural insights. The structure of VP7 comprises two domains: Domain I and Domain II (Fig. 5A and B) (5), both of which contain neutralizing epitopes (5, 10). To determine which domain is important for susceptibility to neutralizing antibodies, we generated viruses harboring chimeric VP7 proteins. Specifically, we exchanged the Domain II regions of the high-susceptibility genotype G1 and the low-susceptibility genotype G33 and generated chimeric viruses. Replication of the chimeras was similar to that of reassortant viruses with the respective Domain I genotypes, suggesting that Domain I may play a key role in viral replication (Fig. 5C).

**TABLE 2:** Susceptibility of VP7 genotypes to neutralization

Classification	Genotype
Susceptible to Rotarix or RotaTeq^*a*^	G1, Equine-like G3, G4, G8, G9, G6, G12, G26, G36
Highly resistant to human sera^*b*^	G2, Bat-like G3, G11, G12, G25, G33
None of the above	Human G3, G5, G13, G14, G27

^*a*^ Neutralization titer >1:100 against either Rotarix- or RotaTeq-immunized serum.

^*b*^ Significantly less susceptible to the human sera than rOdelia (*P* < 0.0001 [Friedman test], and a >1.5 log_2_-fold change in the geometric mean titer).

To analyze susceptibility to neutralization, we used Rotarix-immunized serum and naturally infected human sera; this is because the G1 genotype is highly susceptible to these sera (Fig. 3 and 4). Neutralization tests revealed that the chimeric virus harboring Domain I of G1 was highly susceptible to neutralization, while those harboring Domain I of G33 were less so (Fig. 5D and E). A similar experiment was conducted with the G1 genotype and the low-susceptibility genotype G25. The results were similar, although they were not as clear as those obtained with the G1 and G33 genotypes (Fig. 5F and H). Taken together, the data suggest that Domain I is an important region that determines viral replication and susceptibility to neutralization.

### Single epitope swapping does not affect susceptibility to neutralization

**Fig 6:**
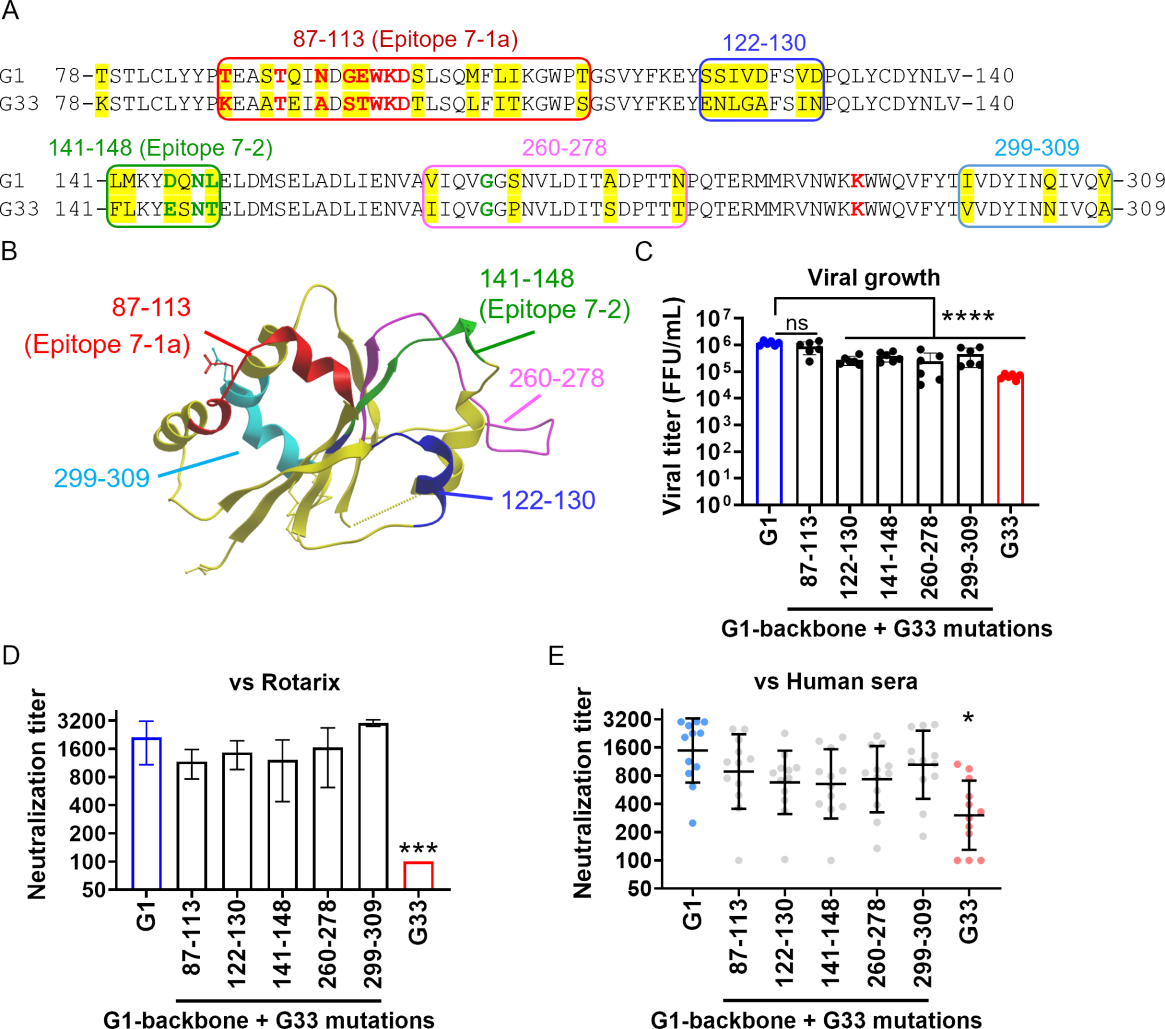
Swapping a single epitope does not alter susceptibility to neutralization. (**A**) Amino acid differences in Domain I of the G1 and G33 genotypes. Differences are highlighted in yellow. Critical residues within the 7-1a and 7-2 epitopes are highlighted in bold red and green, respectively (5). The regions containing these amino acid differences are divided into five sections, highlighted by squares, with the amino acid residue numbers indicated above. (**B**) Structure of Domain I of VP7. Each of the five regions is highlighted. (**C**) Growth of the epitope-swapped viruses. MA104 cells were infected with the viruses at an MOI of 0.001 and harvested at 72 h post-infection. Statistical significance was analyzed using one-way ANOVA with Dunnett’s multiple comparison test. *P* values < 0.05 were considered statistically significant. ****P value < 0.0001. (**D**) Susceptibility of the epitope-swapped viruses to neutralization by Rotarix-immunized serum. Statistical significance was analyzed using one-way ANOVA with Dunnett’s multiple comparison test. *P* values < 0.05 were considered statistically significant and shown in the figure. ***P value < 0.001. (**E**) Susceptibility of the epitope-swapped viruses to neutralization by human sera. Each circle represents serum from an individual (*n* = 12). A substantial reduction in neutralization, denoted by *, was defined by the following criteria: (i) *P* < 0.0001 by Friedman test (compared with the G1 monoreassortant), and (ii) a >1.5 log2-fold change in the geometric mean titer (compared with the G1 monoreassortant).

Domain I contains major neutralizing epitopes, including the 7-1a and 7-2 epitopes (Fig. 5A) (5). Interestingly, we found that amino acid differences between the G1 and G33 genotypes were concentrated within these epitope regions (Fig. 6A and B). Additionally, several amino acid differences were also found outside these epitope regions. To identify which amino acid residues affect susceptibility to neutralization, we focused on five specific regions showing differences between the G1 and G33 genotypes (positions 87–113, 122–130, 141–148, 260–278, and 299–309). Each of these regions from the G33 genotype was substituted individually into the G1 genotype backbone. Although the epitope-swapped viruses were rescued successfully (Fig. 6C), attempts to create reciprocal viruses by swapping G1 regions into the G33 backbone were unsuccessful, even after multiple attempts. We expected that at least one of the recombinant epitope-swapped viruses would show reduced susceptibility to neutralizing antibodies (i.e., a <1.5 log_2_-fold change of geometric mean titer compared with rOdelia); however, none did (Fig. 6D and E). This suggests that swapping a single epitope region is not sufficient to alter susceptibility to neutralization.

## DISCUSSION

Analyzing the effects of rotavirus genotype on neutralization susceptibility is important for predicting future outbreak strains and for developing improved rotavirus vaccines. Here, we successfully established 30 recombinant viruses with distinct genotypes using a human rotavirus backbone. It is important to note that generating recombinant viruses is not always successful. Specifically, the G7, G15, G16, G17, G18, G19, G20, G21, G22, G23, G24, G29, G31, G34, G35, G40, and G41 VP7 genotypes could not be rescued. The degree of rotavirus replication and the virus rescue efficiency depend on complex interactions of the 11 dsRNA segments and their products. As VP4, VP6, and VP7 proteins interact structurally to form the viral particle (7), double-reassortant (VP7 + VP4) or triple-reassortant (VP7 + VP4 + VP6) strains may be rescued through correct interactions. The failure to generate reassortant viruses for certain genotypes may suggest a relatively low likelihood of reassortant viruses emerging from human rotavirus (at least in combination with the Odelia strain). Interestingly, these genotypes appear to form clusters in the phylogenetic tree (Fig. 2B). In particular, avian rotavirus genotypes (i.e., G7, G17, G18, G19, G22, G23, and G40) could not be rescued. Identifying the regions responsible for compatibility with human rotavirus will help predict the potential emergence of reassortant viruses. Alternatively, further improvements to the reverse genetics system may allow for the rescue of genotypes that were not generated successfully in this study (48, 49).

Genotype influences not only susceptibility to neutralization but also viral replication (Fig. 1, 2, 5 and 6). VP4 facilitates viral attachment by interacting with cellular receptors; animal rotaviruses use sialic acid, whereas human rotaviruses utilize histo-blood group antigens (50, 51). VP7 contributes to viral entry by engaging with coreceptors, including integrins and heat shock cognate protein 70 (9, 52). Genotypic variations are likely to affect these functions, resulting in differences in viral replication levels. Indeed, VP4 or VP7 reassortant viruses generally showed lower growth than the parental strain (14, 16), which is consistent with our findings (Fig. 1 and 2). The monoreassortant viruses generated in this study provide a valuable tool for investigating genotype-specific functional differences.

Previously, we generated VP7 monoreassortants using clinical isolates prevalent in the United States and then analyzed their susceptibility to neutralization by vaccinated sera (15). In the present study, we conducted a more comprehensive analysis using Thai clinical isolates, as well as synthesized prototype strains. The results of the two studies differ somewhat, despite using the same vaccinated rabbit sera. For example, the United States G2 genotype was highly susceptible to neutralizing antibodies induced by both the Rotarix and RotaTeq vaccinations, whereas the Thai G2 genotype was not. Although the backbone virus used in these studies differed (simian rotavirus SA11 strain vs human rotavirus Odelia strain, respectively), this highlights the intra-genotype differences between isolates from the United States and Thailand. Intra-genotype differences may explain, at least in part, variations in the efficacy of rotavirus vaccines between high-mortality and low-mortality countries (31), underscoring the need for further studies that generate monoreassortant viruses from clinical isolates from diverse geographical regions. This also emphasizes the importance of developing region- or country-specific rotavirus vaccines that incorporate VP4 and VP7 genes from strains circulating in the target region.

Under our experimental conditions, the G2, bat-like G3, G11, G12, G25, and G33 genotypes were less susceptible to neutralization by antibodies induced by vaccination and natural infection (Fig. 3 and 4). The prevalence of the G2 genotypes in humans is increasing worldwide, both in countries that do and do not have rotavirus vaccination programs (36, 38–40, 53). Our data suggest that, at least for the Thai G2 genotype, there may be reduced susceptibility to neutralizing antibodies induced by vaccination. Notably, 16 amino acid substitutions were identified between the Thai G2 strain and the G2 strain included in RotaTeq, which may influence neutralization susceptibility (Fig. S1). Although neutralizing antibodies alone cannot account fully for protection efficiency, our findings may help explain the rising prevalence of the G2 genotype.

Although the G12 genotype has been increasing worldwide, it has not yet become prevalent in Thailand (43, 44). As a result, the human sera used in this study may lack genotype-specific neutralizing antibodies against the G12 genotype, resulting in low susceptibility of the G12 monoreassortant virus. As observed in other countries (38, 41), the introduction of the G12 genotype into Thailand could potentially lead to an epidemic.

The other genotypes (G11, bat-like G3, G25, and G33) are not commonly prevalent in humans. The G11 genotype originated from pigs (54), and reassortment events involving the G11 gene segment with human rotavirus have been reported (55, 56). Notably, the bat-like G3, G25, and G33 genotypes originated from bats; indeed, several bat-origin rotaviruses may have the potential for zoonotic transmission (23, 44, 57). Although the replication efficiency of these reassortant viruses was lower than that of the parental rOdelia strain (Fig. 2C and D), their ability to evade human immunity is concerning. These genotypes could also have the capacity to cause widespread infection in humans, similar to the G3 equine-like genotype (25, 26). Since the introduction of RotaTeq in Thailand in 2005 and Rotarix in 2008 (both were incorporated into the national immunization program in 2020), genotype distributions have shifted, showing increased genetic diversity (58). Taken together, data suggest that monitoring the emergence of these strains is crucial for predicting and preventing future rotavirus outbreaks.

In addition to continuous monitoring, the development of highly effective rotavirus vaccines is essential to prevent future outbreaks. Our data suggest that the VP7 genotype, rather than the VP4 genotype, has a greater impact on susceptibility to neutralizing antibodies (Fig. 3 and 4). The chimeric virus experiments revealed that Domain I of VP7 is a key determinant of susceptibility to neutralization, although Domain II, particularly in the G25 genotype, may also contribute to this susceptibility (Fig. 5). These findings indicate that the VP7 Domain I is a promising target for designing vaccine antigens. This also suggests that antibodies targeting Domain I might be induced to a greater extent than those targeting Domain II. This hypothesis needs to be verified through studies involving depletion of Domain II antibodies by mixing sera with soluble Domain II. Although Domain I contains several neutralizing epitopes (5), we could not identify a specific epitope region responsible for susceptibility. This suggests that multiple epitopes in Domain I may contribute to susceptibility to neutralization, thereby highlighting the complexity of the immune response to rotavirus. Further experiments to narrow down the specific region responsible for reduced susceptibility would be of interest.

One limitation of this study is the use of intramuscularly immunized rabbit sera instead of sera from orally vaccinated humans for the neutralization tests, as well as the use of only one rabbit per vaccine, which may not fully capture antibody variability. Future studies should consider including sera from multiple orally immunized rabbits or, ideally, from vaccinated humans to validate and strengthen these results. In particular, using sera from infants who were either vaccinated or naturally infected would more accurately reflect the nature of neutralizing antibody responses. While adult sera were used in this study due to practical constraints, they still provide useful insights into population-level immune exposure.

Another limitation is that serum neutralizing antibody titers do not always correlate with protection efficacy. Indeed, protection efficacy often exceeds the seroconversion rate (59, 60). In addition to neutralizing antibodies, non-neutralizing antibodies targeting VP6, as well as mucosal IgA levels, are important protective factors (61, 62). Further research into immunological correlates of protection may be facilitated by recent advances in the murine rotavirus model (63, 64). Notably, antibodies induced by Rotarix immunization did not neutralize viruses harboring most of the VP7 genotypes in our experiments (Fig. 3); however, rotavirus vaccinations provide cross-genotype protection against non-vaccine genotypes (65, 66). For example, Rotarix vaccination confers protection against the G2P[4] genotype for at least 2 years (66), emphasizing the importance of factors beyond neutralizing antibodies. Even though the serum neutralizing antibody titer is a primary determinant of protection (67–69), the results should be interpreted with caution.

In conclusion, we identified rotavirus genotypes that are less susceptible to neutralization by antibodies induced by vaccination or natural infection. These genotypes may have the potential to reassort with human rotavirus and evade pre-existing immunity, potentially causing outbreaks in humans. In particular, bat-origin genotypes, including bat-like G3, G25, and G33, need to be monitored carefully. To prevent future outbreaks, designing next-generation vaccines based on Domain I of the VP7 protein is a promising strategy. The findings from this study may make a crucial contribution to the prevention of future rotavirus outbreaks.

## MATERIALS AND METHODS

### Cells

Monkey kidney MA104 cells were cultured in Dulbecco’s modified Eagle’s medium (DMEM; Nacalai Tesque) supplemented with 5% fetal bovine serum (FBS; Gibco). Baby hamster kidney cells (BHK-T7) stably expressing T7 RNA polymerase were cultured in DMEM supplemented with 5% FBS and 1 µg/mL puromycin (Sigma-Aldrich). All cell cultures were maintained at 37°C/5% CO_2_.

### Viruses

The human rotavirus Odelia strain was used as the backbone virus. Recombinant Odelia strain was generated as described previously and is referred to herein as rOdelia (47). rOdelia strains harboring the VP4 or VP7 segments from other rotavirus strains (monoreassortant viruses) were also generated as described previously (14, 15). rOdelia and monoreassortant viruses were propagated in MA104 cells cultured in DMEM supplemented with 0.5 µg/mL trypsin (Sigma-Aldrich). The propagated viruses were used directly for viral titration or neutralization tests without prior activation by trypsin. Recombinant viruses were used for experiments at passage number 2 or 3.

### Antibodies

Rabbit anti-NSP4 antiserum was prepared by immunizing rabbits with a synthetic peptide spanning amino acid residues 158–171 of the rotavirus NSP4 protein (Eurofins Genomics) (70). Additionally, rabbit antisera were prepared by immunizing rabbits two times (intramuscularly) with the Rotarix or RotaTeq vaccines (15).

### Clinical samples

Human fecal samples were collected in Chiang Mai, Thailand, between 2010 and 2020 (18, 43–45). RNA was extracted from the samples, and RT-PCR was performed to amplify the VP4 and VP7 genes (14). The resulting amplicons were cloned into viral rescue plasmids (see below) and sequenced. The study was approved by the ethics committees of the Faculty of Medicine, Chiang Mai University, Thailand (approval number MIC-2557-02710), and the Research Institute for Microbial Diseases, Osaka University, Japan (approval number 2023-17). Sequence data for several strains have been deposited in GenBank (44, 45). The sequence data obtained in this study have been deposited in GenBank under accession numbers LC853150–LC853217, combining ID numbers and strain names.

Serum samples from 12 healthy volunteers were collected in Bangkok, Thailand. The volunteers (aged 31–58 years) included 6 males and 6 females. The study was approved by the ethics committees of Bangkok, Thailand (approval number, E10h/64_EXP), the Ministry of Public Health, Thailand (approval number, 1/2564), the Research Institute for Microbial Diseases, Osaka University, Japan (approval number, 2020-20), and the Research and Education Promotion Foundation, Thailand (approval number, REP22-01).

### Plasmids

Viral rescue plasmids containing each rotavirus genome segment flanked by the T7 promoter and HDV ribozyme-T7 terminator were used to rescue rOdelia. These plasmids were named pT7-VP1-Odelia, pT7-VP2-Odelia, pT7-VP3-Odelia, pT7-VP4-Odelia, pT7-VP6-Odelia, pT7-VP7-Odelia, pT7-NSP1-Odelia, pT7-NSP2-Odelia, pT7-NSP3-Odelia, pT7-NSP4-Odelia, and pT7-NSP5-Odelia (47). Additionally, pCAG vector plasmids containing rotavirus NSP2 or NSP5, or vaccinia virus D1R or D12L, were used; these plasmids were named pCAG-NSP2SA11, pCAG-NSP5SA11, pCAG-D1R, and pCAG-D12L, respectively (42, 71).

To rescue monoreassortant viruses, rescue plasmids containing the VP4 or VP7 segments of other rotavirus strains were constructed. The rescue plasmids used for the Thai clinical isolates were prepared as described previously (14), while the rescue plasmids used for prototype strains were synthesized (Genscript). Regarding prototype strains lacking sequence information related to untranslated regions, these were completed using sequences from the Odelia strain. Plasmids containing chimeric VP7 were constructed using the NEBuilder HiFi DNA Assembly Cloning Kit (New England Biolabs) and validated by Sanger DNA sequencing.

### Phylogenetic analysis

The VP4 or VP7 genes of prototype strains and vaccine strains were retrieved from GenBank, and the sequences were aligned using Genetyx ver. 16 software (Genetyx). Neighbor-joining trees were constructed using a Kimura two-parameter model in MEGA11 software. Bootstrap values (1,000 replicates) were calculated and reported for relevant nodes on a representative tree. Only bootstrap values > 90 are shown in the figures.

### Generation of recombinant rotaviruses

rOdelia was generated as described previously (47). Briefly, BHK-T7 cells were seeded into a 12-well plate at a density of 1 × 10⁵ cells per well. On the following day, the BHK-T7 cells were co-transfected with 11 viral rescue plasmids (pT7-VP1-Odelia, pT7-VP2-Odelia, pT7-VP3-Odelia, pT7-VP4-Odelia, pT7-VP6-Odelia, pT7-VP7-Odelia, pT7-NSP1-Odelia, pT7-NSP2-Odelia, pT7-NSP3-Odelia, pT7-NSP4-Odelia, and pT7-NSP5-Odelia) along with 4 plasmids that enhance rescue efficiency (pCAG-NSP2SA11, pCAG-NSP5SA11, pCAG-D1R, and pCAG-D12L).

At 24 h post-transfection, MA104 cells suspended in DMEM containing 0.5 µg/mL trypsin were added to the BHK-T7 cells and co-cultured for at least 5 days. The cells were then lysed by freeze-thawing and transferred to a new monolayer of MA104 cells for further incubation for 5 days. Successful rescue of viruses was confirmed by the presence of infectious virions in a viral titration assay (see below).

To generate monoreassortant viruses, the plasmid pT7-VP4-Odelia or pT7-VP7-Odelia was replaced with a plasmid containing the VP4 or VP7 segment, respectively, from other rotavirus strains. In the initial trial, four wells in a 12-well plate were used to rescue each recombinant virus. If the rescue of a certain genotype failed, the experiment was repeated using 12 wells in a 12-well plate. Failure to rescue a recombinant virus was defined as failure in both trials.

### Virus titration

Viral titration was performed as described previously (72). Briefly, MA104 cells were seeded in 96-well plates at a density of 1 × 10⁴ cells per well. On the following day, serially diluted rotavirus samples were inoculated onto the cell monolayers and incubated overnight at 37°C. The cells were then fixed for 30 min with 4% formaldehyde and permeabilized for 15 min with 0.5% Triton-X. The cells were then blocked for 1 h in PBS containing 2% FBS. Next, the cells were incubated for 1 h at room temperature with an anti-rotavirus NSP4 antibody, followed by a 1 h incubation at room temperature with goat anti-rabbit IgG Alexa Fluor 488 (Thermo Fisher). After each incubation, the cells were washed three times with PBS. Nuclei were stained with Hoechst (Thermo Fisher). Infected cells were counted manually under an Axio Observer 7 fluorescence microscope (Zeiss), and the viral titer was expressed as focus-forming units (FFU) per mL.

### Virus replication assay

The assay was conducted as described previously (72). Briefly, MA104 cells were seeded into 24-well plates at a density of 7.5 × 10⁴ cells per well. The cells were then infected with viruses at a multiplicity of infection (MOI) of 0.001 and incubated for 1 h at 37°C. Next, the infected cells were washed two times with PBS, followed by the addition of DMEM containing 0.5 mg/mL trypsin. The cells were then incubated for 72 h prior to three cycles of freeze-thawing. The resulting cell lysates were used for the viral titration assay.

### Neutralization test

MA104 cells were seeded into a 96-well plate at a density of 1 × 10⁴ cells per well. On the following day, serially diluted antibody was mixed with about 200 FFU of rotavirus and incubated for 1 h at 37°C, followed by inoculation onto the seeded cells. On the following day, the cells were fixed, and infected cells were visualized following the viral titration method described earlier. Infected cells were counted using the Cytation 5 imaging system (Agilent), and the percentage neutralization was calculated based on the number of foci.

### Statistical analysis

Data analysis was performed using GraphPad Prism 9 (GraphPad Software, Inc.). Data are expressed as the mean ± standard deviation from at least two independent experiments.

## SUPPLEMENTAL MATERIAL

Supplemental material mbio.00897-25-s0001.docx Fig S1 and S2;Table S1.

ASM does not own the copyrights to Supplemental Material that may be linked to, or accessed through, an article. The authors have granted ASM a non-exclusive, world-wide license to publish the Supplemental Material files. Please contact the corresponding author directly for reuse.
