# Supplementary material for: A rotavirus VP4 or VP7 monoreassortant panel identifies genotypes that are less susceptible to neutralization by systemic antibodies induced by vaccination or natural infection
Source: mBio. 2025 May 30;16(7):e00897-25. doi: 10.1128/mbio.00897-25 (PMC12239578; doi:10.1128/mbio.00897-25)
Supplement: Supplemental material — Fig S1 and S2;Table S1. [file mbio.00897-25-s0001.docx]

**Supplemental material**

**A rotavirus VP4 or VP7 monoreassortant panel identifies genotypes that are less susceptible to neutralization by systemic antibodies induced by vaccination or natural infection**

Tomohiro Kotaki^1^, Yuta Kanai^1^, Kristen M. Ogden^2^, Megumi Onishi^1^, Kattareeya Kumthip^3,4^, Pattara Khamrin^3,4^, Patcharaporn Boonyos^5^, Pornkamol Phoosangwalthong^5^, Phakapun Singchai^6^, Tipsuda Luechakham^6^, Shohei Minami^1^, Zelin Chen^1^, Katsuhisa Hirai^1^, Ratana Tacharoenmuang^6^, Hiroto Mizushima^5^, Hiroshi Ushijima^7^, Niwat Maneekarn ^3,4^, Takeshi Kobayashi^1,8,9*^

^1^Department of Virology, Research Institute for Microbial Diseases, The University of Osaka, Osaka, Japan.

^2^Department of Pediatrics, Vanderbilt University Medical Center, Tennessee, United States.

^3^Department of Microbiology, Faculty of Medicine, Chiang Mai University, Chiang Mai, Thailand.

^4^Center of Excellence in Emerging and Re-emerging Diarrheal Viruses, Chiang Mai University, Chiang Mai, Thailand.

^5^Thailand-Japan Research Collaboration Center on Emerging and Re-emerging Infections, Nonthaburi, Thailand.

^6^Department of Medical Sciences, Ministry of Public Health, Nonthaburi, Thailand.

^7^Department of Pathology and Microbiology, Nihon University School of Medicine, Tokyo, Japan.

^8^Center for Infectious Disease Education and Research, The University of Osaka, Osaka, Japan.

^9^Center for Advanced Modalities and DDS, The University of Osaka, Osaka, Japan.

***Corresponding author:**

Takeshi Kobayashi

Research Institute for Microbial Diseases, The University of Osaka,

3-1 Yamadaoka, Suita, Osaka 565-0871, Japan

Phone no: 81-6-6879-8335

E-mail Address: tkobayashi@biken.osaka-u.ac.jp

**
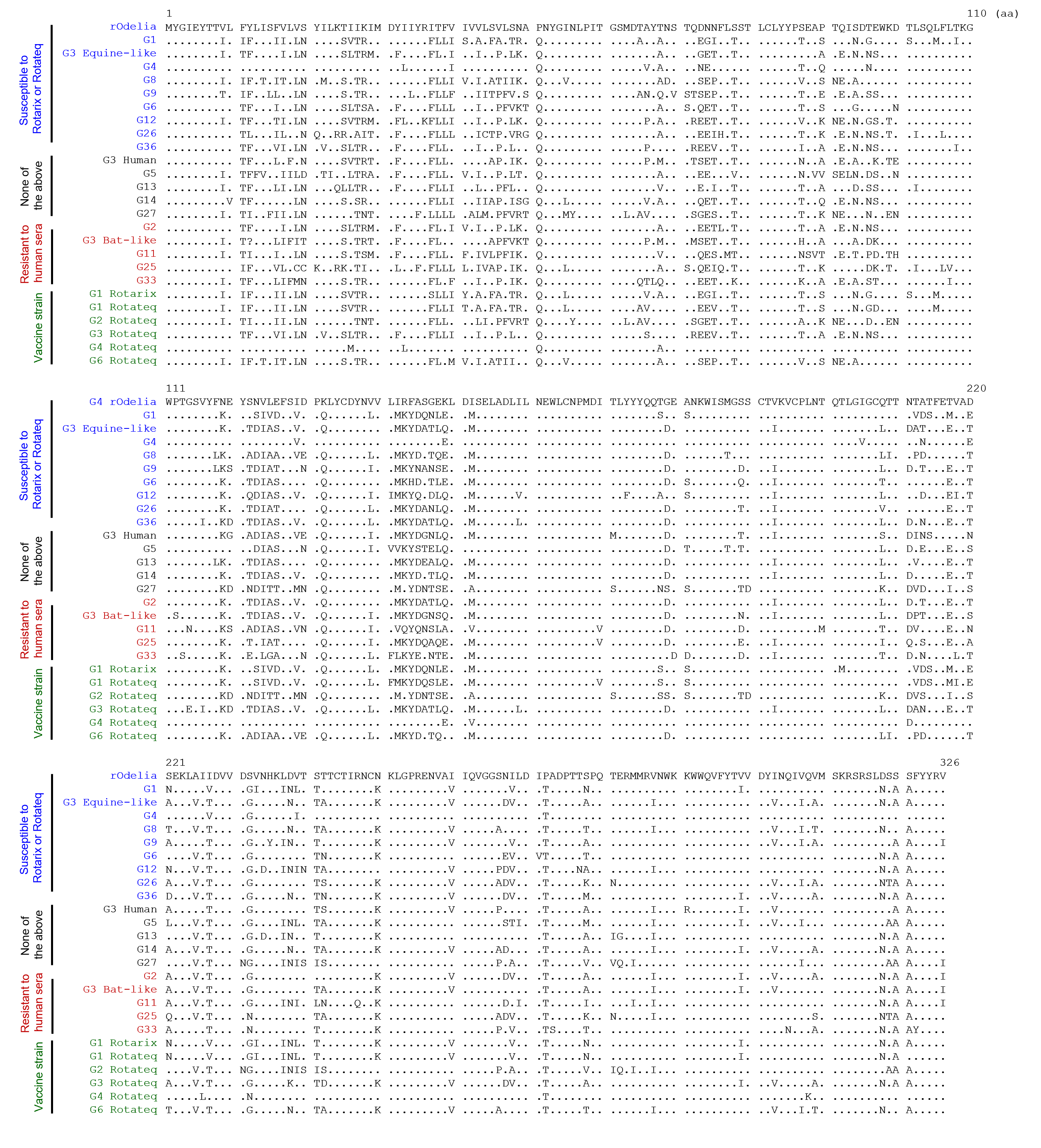
**

**Supplementary Figure S1. Amino acid sequence alignment of the VP7 genotypes used in the neutralization test.**

The genotypes were classified into four groups (see Table 2).


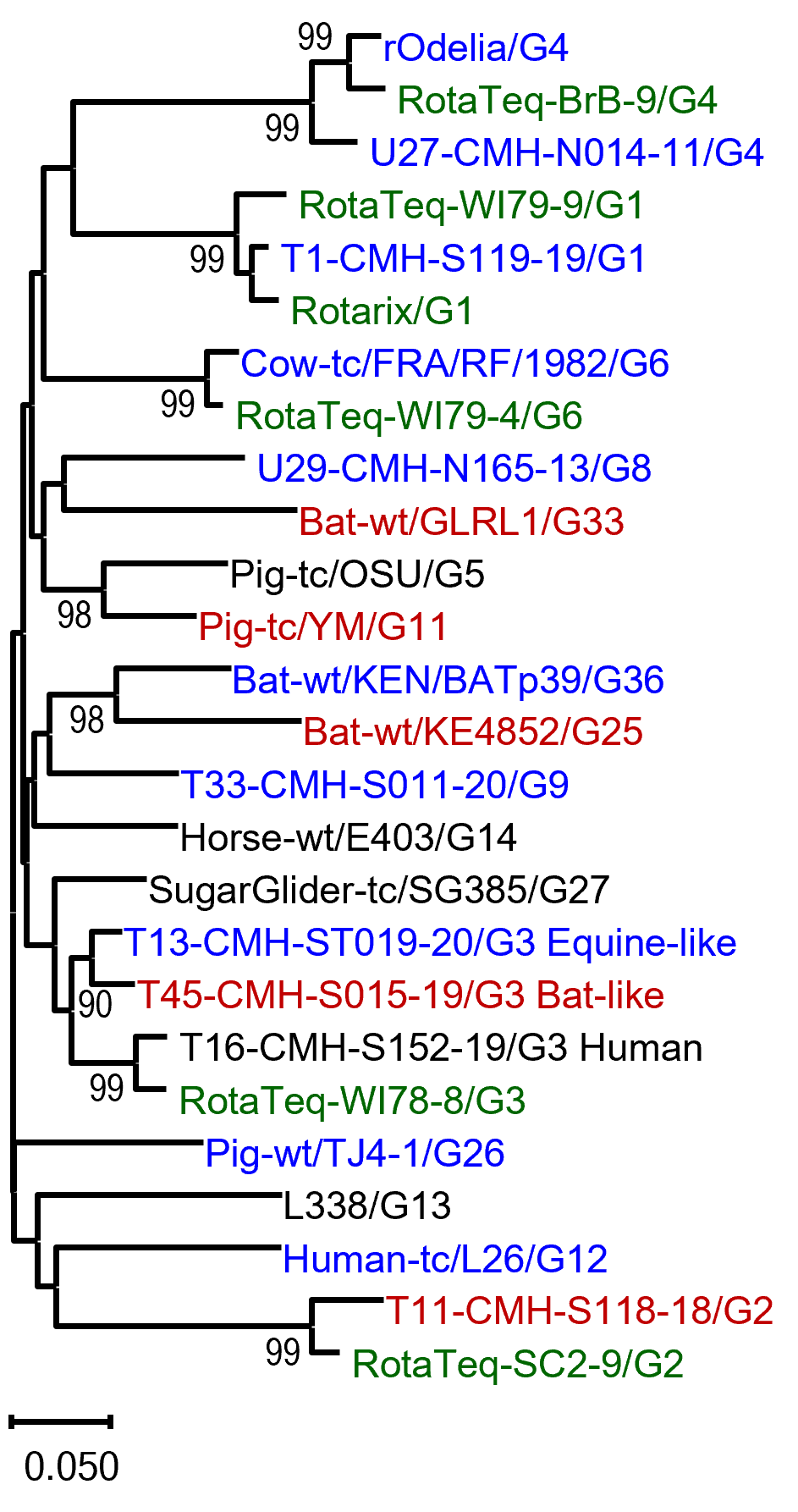


**Supplementary Figure S2. Phylogenetic analysis of the VP7 genotypes used in the neutralization tests.**

Amino acid sequences used for the analysis. The genotypes were classified into four groups (see Table 2). Blue, red, green, and black indicate genotypes susceptible to Rotarix or RotaTeq, genotypes highly resistant to human sera, vaccine strains, and those not falling into any of the above categories, respectively.

**Supplementary Table S1. Information on the viral sequence data used for the rescue of monoreassortant viruses**

| Source | Protein | Genotype | Strain name | Origin | Accession | Virus rescue* |
| --- | --- | --- | --- | --- | --- | --- |
| Clinical samples | VP4 | P[8] cluster 1 | T25-CMH-S022-18 | Human | LC853166 | Successful |
|  |  | P[8] cluster 2 | T41-CMH-S069-18 | Human | LC853179 | Successful |
|  |  | P[8] cluster 3 | T20-CMH-ST230-19 | Human | LC853161 | Successful |
|  |  | P[8] cluster 4 | T21-CMH-R035-19 | Human | LC853162 | Successful |
|  |  | P[8] cluster 5 | T16-CMH-S152-19 | Human | LC853160 | Successful |
|  |  | P[4] | T9-CMH-S006-20 | Human | LC853154 | Successful |
|  |  | P[19] | U30-CMH-S070-13 | Human | LC853184 | Successful |
|  |  | P[6] | U27-CMH-N014-11 | Human | LC853182 | Successful |
|  | VP7 | G1 | T1-CMH-S119-19 | Human | LC853185 | Successful |
|  |  | G2 | T11-CMH-S118-18 | Human | LC853189 | Successful |
|  |  | G3 Equine-like | T13-CMH-ST019-20 | Human | LC853190 | Successful |
|  |  | G3 Bat-like | T45-CMH-S015-19 | Human | LC853214 | Successful |
|  |  | G3 Human | T16-CMH-S152-19 | Human | LC853193 | Successful |
|  |  | G4 | U27-CMH-N014-11 | Human | LC853215 | Successful |
|  |  | G8 | U29-CMH-N165-13 | Human | LC853216 | Successful |
|  |  | G9 | T33-CMH-S011-20 | Human | LC853204 | Successful |
| Gene synthesis | VP4 | P[20] | RVA/Mouse-tc/XXX/EHP/1981/G16P[20] | Mouse | U08424 | Successful |
|  |  | P[25] | RVA/Human-wt/BGD/Dhaka6/2001/G11P[25] | Human | GU199520 | Failed |
|  |  | P[31] | RVA/Chicken-tc/DEU/06V0661/2006/G19P[31] | Chicken | EU486962 | Failed |
|  |  | P[32] | RVA/Pig-wt/IRL/61-07-ire/2007/G2P[32] | Pig | FJ492835 | Failed |
|  |  | P[39] | RVA/CommonGull-wt/JPN/Ho374/2013/G28P[39] | Common Gull | LC088221 | Failed |
|  |  | P[43] | RVA/Bat-wt/CMR/BatLy03/2014/G25P[43] | Bat | KX268779 | Successful |
|  |  | P[48] | RVA/Bat-wt/CHN/GLRL1/2005/G33P[48] | Bat | KX814933 | Failed |
|  |  | P[49] | RVA/Pig-wt/BGD/214016006/2014/G9P[49] | Pig | KY905314 | Failed |
|  |  | P[51] | RVA/Bat-wt/KEN/BATp39/2015/G36P[51] | Bat | MH285840 | Successful |
|  | VP7 | G5 | RVA/Pig-tc/USA/OSU/1977/G5P9[7] | Pig | KR052772 | Successful |
|  |  | G6 | RVA/Cow-tc/FRA/RF/1982/G6P[1] | Cow | X65940 | Successful |
|  |  | G7 | RVA/Turkey-tc/IRL/Ty-3/1979/G7P[17] | Turkey | AB080737 | Failed |
|  |  | G11 | RVA/Pig-tc/MEX/YM/1983/G11P9[7] | Pig | M23194.1 | Successful |
|  |  | G12 | RVA/Human-tc/PHL/L26/1987/G12P[4] | Human | M58290.1 | Successful |
|  |  | G13 | RVA/Horse/L338/G13P[18] | Horse | KR086412 | Successful |
|  |  | G14 | RVA/Horse-wt/ARG/E403/2006/G14P[12] | Horse | JF712582 | Successful |
|  |  | G15 | RVA/Cow-wt/ARG/B383/1998/G15P[11] | Cow | FJ347116 | Failed |
|  |  | G16 | RVA/Mouse-tc/XXX/EHP/1981/G16P[20] | Mouse | U08425 | Failed |
|  |  | G16 | RVA/Mouse-tc/ETD822/G16P[16] | Mouse | GQ479955 | Failed |
|  |  | G17 | RVA/Turkey-tc/IRL/Ty-1/1979/G17P[17] | Turkey | L01098 | Failed |
|  |  | G18 | RVA/Pigeon-tc/JPN/PO-13/1983/G18P[17] | Pigeon | D82979 | Failed |
|  |  | G19 | RVA/Avian/Ch-1/G19P[17] | Avian | AB080738 | Failed |
|  |  | G20 | RVA/Human-wt/ECU/Ecu534/2006/G20P[28] | Human | EU805775 | Failed |
|  |  | G20 | RVA/Human-wt/SUR/2014735512/2013/G20P[28] | Human | KX257405 | Failed |
|  |  | G21 | RVA/Cow-wt/JPN/Azuk-1/2006/G21P[29] | Cow | AB454421 | Failed |
|  |  | G21 | RVA/Cow/JPN/CACC/1998/G21P[29] | Cow | AB486011 | Failed |
|  |  | G22 | RVA/Turkey-tc/DEU/03V0002E10/2003/G22P[35] | Turkey | EU486973 | Failed |
|  |  | G23 | RVA/Pheasant-wt/HUN/Phea14246/2008/G23P[x] | Pheasant | FN393054 | Failed |
|  |  | G24 | RVA/Cow-tc/JPN/Dai-10/2007/G24P[33] | Cow | AB513837 | Failed |
|  |  | G25 | RVA/Bat-wt/KEN/KE4852/07/2007/G25P[6] | Bat | GU983676 | Successful |
|  |  | G26 | RVA/Pig-wt/JPN/TJ4-1/2010/G26P[?] | Pig | AB605258 | Successful |
|  |  | G27 | RVA/SugarGlider-tc/JPN/SG385/2012/G27P[36] | Sugar Glider | AB621363 | Successful |
|  |  | G29 | RVA/Human-wt/BEL/BEF06018/2014/G29P[41] | Human | KU128897 | Failed |
|  |  | G29 | RVA/Buffalo-wt/ZAF/4426/2002/G29P[14] | Buffalo | MT234364 | Failed |
|  |  | G31 | RVA/Bat-wt/CMR/BatLi08/2014/G31P[42] | Bat | KX268770 | Failed |
|  |  | G31 | RVA/Bat-wt/ZMB/ZFB14-52/2014/G31P[x] | Bat | LC277161 | Failed |
|  |  | G33 | RVA/Bat-wt/CHN/GLRL1/2005/G33P[48] | Bat | KX814932 | Successful |
|  |  | G34 | RVA/Raccoon-wt/JPN/Rac-311/2011/G34P[17] | Raccoon | LC208550 | Failed |
|  |  | G35 | RVA/Alpaca-wt/PER/Alp11B/2010/G35P[50] | Alpaca | KY971977 | Failed |
|  |  | G36 | RVA/Bat-wt/KEN/BATp39/2015/G36P[51] | Bat | MH285845 | Successful |
|  |  | G40 | RVA/JungleCrow-wt/JPN/JC-105/2019/G40P[56] | Jungle Crow | LC634427 | Failed |
|  |  | G41 | RVA/MultimammateMouse-wt/ZMB/MpR12/2012/G41P[57] | Mouse | LC638706 | Failed |

*Failure was designated if the virus could not be rescued in two independent trials. See Materials and Methods for details.
